# Household Pharmaceutical Waste Disposal as a Global Problem—A Review

**DOI:** 10.3390/ijerph192315798

**Published:** 2022-11-27

**Authors:** Justyna Rogowska, Agnieszka Zimmermann

**Affiliations:** Division of Medical and Pharmacy Law, Department of Social Medicine, Faculty of Health Sciences, Medical University of Gdańsk, Tuwima Str. 15, 80-210 Gdańsk, Poland

**Keywords:** household waste, pharmaceutical waste, disposal of pharmaceutical waste, management of pharmaceutical waste, consumer behavior

## Abstract

The negative effect of the pharmaceuticals presence (persistence?) in various components of the environment is a global problem today. These compounds are released into the environment as a result of, inter alia, their use and improper disposal. Therefore, it is important to reduce excessive drug consumption and to develop a system for the collection of unused/expired pharmaceuticals. The effectiveness of actions in this area is inextricably linked with the need to educate society on how to deal properly with unwanted medications. The aim of the study was to show that the inappropriate handling of unused/expired drugs by society is an important problem in waste management systems, and it impacts the state of the environment. Forty-eight scientific articles published between 2012 and 2021 were taken into account that discussed the systems in various countries for the collection of unused/expired pharmaceuticals. This literature review shows that the main method of disposing of unused/expired medications, according to respondents from different countries, is either by disposing of them in household waste or flushing them into the sewage system. This is also the case in countries with systems or programs for the return of redundant drugs, which indicates that these systems are not sufficiently effective. This may be influenced by many factors, including the lack or ineffective education of the society.

## 1. Introduction

Developments in technology and progress in medicine have resulted in an increase in life expectancy. According to World Health Organization (WHO) data, life expectancy increased from 66.79 to 73.31 during 2000–2019 [1], and from 1900 to 2019, the global average life expectancy more than doubled [2]. On the other hand, social, economic and cultural changes have influenced the development of civilization diseases such as obesity, diabetes, cardiovascular diseases, cancer and autoimmune diseases, which is reflected in the statistics of the consumption of specific categories of pharmaceuticals. According to data of the Organization for Economic Co-operation and Development (OECD), in the years 2000–2017, the consumption of antihypertensive drugs increased by 70% on average, cholesterol-lowering agents threefold and the consumption of antidiabetic drugs double, as with antidepressants [3]. An increase in the use of ‘over-the-counter’ (OTC) medicines was also observed. This is due to the fact that OTC drugs are easily available and affordable [4].

A survey conducted by Hedendrud et al. (2019) in Sweden showed that, in total, 87% of respondents reported the use of OTC drugs in the prior 6-month period [5]. Similar data were obtained by Vatovec et al. (2021) in a study conducted among 421 respondents from the US in which 85% of respondents had obtained OTC drugs in the previous 12 months [6]. In turn, in a study carried out in 2008–2011 on a group of 7091 people in Germany, 40.2% of respondents admitted that in the 7 days preceding the survey, they had used medicines or dietary supplements such as vitamins or minerals [7]. Research conducted by Rogowska et al. (2019) showed that almost 60% of respondents buy OTC pharmaceuticals before they are needed. The same study indicated that analgesics are the most used OTCs (71.8% of respondents) [8]. Similar results were presented by Zorpas et al. (2018). Among 184 respondents living in the district of Nicosia (Cyprus), 65.8% of them most often used painkillers [9].

Furthermore, the COVID-19 pandemic has caused an increase in the consumption of some OTCs (for example, ivermectin is sold as an OTC drug in some countries), and thereby self-medication has become a serious problem [10]. An online survey study was conducted by Quispe-Cañari et al. (2021) [11] on a group of 3792 respondents from Peru. Their objective was to investigate the prevalence of self-medication drugs used for respiratory symptoms in the prevention of COVID-19. The survey showed that the majority of respondents self-medicated with acetaminophen and ibuprofen in the case of preventive use, in the presence of symptoms and for confirmed cases [11].

Antibiotics are a group of pharmaceuticals the excessive consumption of which is of concern. These drugs are used for human and animal disease treatment, growth promotion and prophylaxis [12]. The global consumption of antibiotics increased by 65% between 2000 and 2015 [13]. The effect of the increased consumption of these drugs is increases in their presence in the environment, which may affect the survival, reproduction, metabolism and population of organisms and change the community structure and ecological function of the ecosystem, including biomass production and biodiversity [14]. Furthermore, the overuse of antibiotics is a major driver of antibiotics resistance. Some antibiotics are easily degraded, such as penicillin, whereas others are considerably more persistent, such as fluoroquinolones and tetracyclines; thus, they prevail for a longer time in the environment, spreading to larger distances and accumulating in higher concentrations [15].

The presence of pharmaceuticals in the environment is a consequence of both their usage and disposal. The main source of pharmaceuticals is the excretion of active substances consumed by humans and animals via urine and feces (between 30 and 90% of the orally administered dose is excreted as active substances in the urine of humans and animals) and the incorrect disposal of unused medical products into toilets and sinks or incorrect disposal via solid waste [16]. By limiting the excessive consumption of drugs, especially OTCs, or by placing emphasis on the development of more effective methods of wastewater treatment, the release of pharmaceutical residues (unaltered or as metabolites) that pass through the body into the sewage systems may be reduced. Moreover, the incorrect disposal of unused medical products can be minimized through the development and/or improvement of take-back schemes and awareness-raising amongst the public.

However, it seems that there is no global strategy for limiting the production and disposal of pharmaceutical waste. On the other hand, the problem of the improper handling of unnecessary drugs by common people is a very important issue. For example, according to the report published by UNESCO/HELCOM (United Nations Educational, Scientific and Cultural Organization/Baltic Marine Environment Protection Commission), wastewater is the main source of pollution of the Baltic Sea with pharmaceuticals [16]. At the same time, wastewater treatment plants located around the Baltic Sea are not designed to remove micro-pollutants, and their modernization will be a long and costly process. Therefore, the proper disposal of unnecessary drugs by the inhabitants of the Baltic Sea region is crucial to reduce pharmaceutical content in the Baltic Sea environment. Moreover, both in the European Union and within HELCOM, it has been indicated that one of the activities aimed at limiting pharmaceuticals in the environment is to improve unnecessary drug collection systems within the society and increase public awareness of these systems, including their purpose and environmental effects resulting from improper drug handling. These actions were included in the European Union Strategic Approach to Pharmaceuticals in the Environment, a European One Health Action Plan against Antimicrobial Resistance and the updated Baltic Sea Action Plan [17,18,19].

In the view of the above, the aim of the study is to show that the inappropriate handling of unused/expired drugs by society is an important problem in waste management systems, and it impacts the state of the environment. The publication also focuses on the actions taken to reduce the impact of pharmaceutical waste on the environment, which are already implemented in various countries.

## 2. Methods

This review was based on 48 articles published between 2012 and 2021, obtained from databases such as Scopus, Google Scholar and PubMed, and the references contained in these articles. In order to retrieve articles from databases, keywords (and their combinations) such as ‘disposal of pharmaceutical waste’, ‘drug disposal’ and ‘household medical waste’ were entered in the search field. This period of time was selected to include in the review countries from different continents and with different levels of socioeconomic development.

## 3. Pharmaceutical Waste

Around the world, the definition of waste is different in the legal systems of individual countries [20]. In the EU, according to Directive 2008/98/EC of the European Parliament and of the Council of 19 November 2008 on waste, and repealing certain Directives (OJ L 312 22.11.2008, p. 3), ‘waste’ means any substance or object that the holder discards or intends or is required to discard. Pharmaceutical waste seems to be both expired drugs and unused drugs. Unused drugs might be used by other patients; however, in view of safety reasons (there is a worldwide problem with falsified drugs), such drugs can be offered only by a licensed pharmacy. The main reasons why a drug becomes unwanted by a patient or eventually remains unused are:

- Changing the dosage of the drug or changing the drug;
- The death of the patient;
- The noncompletion of therapy or inappropriate use of drugs by the patient (especially antibiotics);
- The discontinuation of therapy due to side effects [21].

Sasu et al. (2012) showed that more than half (59%) of the respondents from Ghana finished their medication, and the remaining respondents stopped taking medicines when they thought they felt better [22]. Ayele and Mamu (2018) indicated that the reasons given by Ethiopian respondents for no longer using drugs were mostly due to recovery from/improvement in the disease or symptoms (53.3%) and forgetting to take the drugs (16.7%) [23]. Respondents from Malaysia admitted that they had unused medicines due to the fact that they stopped taking medicines when they felt better (76.9%), their doctor changed their treatment (50.3%), they experienced unwanted side effects (49%), they did not take the medicines as instructed/prescribed (47.2%) and they did not feel better after taking the medicines (46%) [24]. Another survey, carried out by Hassali and Shakeel (2020), indicated that the main reasons why the respondents keep unused medications at home were that their treatment was changed by their doctor (28.6%) and they felt better (25.1%). Furthermore, respondents kept unused medications for future use (23.9%) [25]. The main reasons why respondents from Tanzania stopped taking medications were, above all, recovering from their illness prior to completing treatment (82.2%), intolerable side effects of the medications (8.3%), a change in the treatment regime (6.2%) and forgetting to take the medications (3.3%) [26]. Similar results were obtained in studies conducted among the inhabitants of Indonesia. The majority of the respondents revealed that the reason they were no longer taking the drug was because their health had improved (82.7%, 97%, depending on the study). Other reasons were: a change in medication by a doctor, no therapeutic effects according to the respondents, alterations in the prescription, experiencing side effects and a switch to herbal remedies [27,28]. Research by Vatovec et al. (2017) on a group of American students indicated one more important reason for having leftover OTC drugs—too many in the package in relation to needs [29].

## 4. Collection System of Unused/Expired Pharmaceuticals

Methods for residents to dispose of unused/expired medications depend on socioeconomic culture as well as legal regulations and regulatory guidelines [30]. In many countries, there are no legal powers indicating the entities responsible for collecting unwanted drugs from residential areas. The provisions mainly concern unused or expired drugs from health care institutions and pharmacies but not from households. Collection systems are organized at national, regional or local levels; the system costs may be concentrated on one entity or spread between different entities [16]. Pharmacies operating in such systems, on a mandatory or voluntary basis, play an important role in the collection of unwanted medicines from patients.

### 4.1. Low and Middle-Income Countries

In low- and middle-income countries, there are often no guidelines regarding the disposal of medications by the public. In Ethiopia, there is no national policy that aims to control the safe disposal of unused/expired medicines or raise public awareness of this issue [23]. Similarly, in Tanzania, the only existing guideline established by the Tanzania Medicines and Medical Devices Authority (TMDA) is for the disposal of expired or unused medications in drug dispensing units, health centers and hospitals [26]. It is the same case in Malaysia, where there are laws only regarding hazardous waste from health care facilities and pharmaceutical companies. According to the law, a waste producer is obliged to appoint a licensed contractor who will collect the waste for disposal at scheduled waste incinerators or secure landfills [31]. Unfortunately, these provisions do not apply to household pharmaceutical waste, which, in effect, results in pharmaceuticals that are discarded in household waste being disposed of at landfills. However, in Malaysia, there is a voluntary medicine take-back program [31]. In India, unused and expired antibiotics are categorized as hazardous waste, and they should be collected separately from other household waste, but this is not adhered to, and in general, the public is not aware of these rules and regulations [32]. In Brazil, despite the fact that the regulations on the handling of medical waste appeared in the 1990s, it was only in 2020 that regulations regarding the collection of unnecessary drugs from residents appeared. In 1993, regulations (CONAMA Resolution No. 5) were implemented in Brazil according to which drug residues were classified as group B, which may not only pose a threat to the environment but also to public health [33]. Then, in 2004, we implemented a Waste Management Plan of Health Services that related to the management, treatment and final disposal of waste but only in the health services [33,34]. Introduced in 2010, the National Solid Waste Policy did not regulate household pharmaceuticals. In 2020, Decree N° 10.388 was implemented, which provides a return system for expired or unused household medicines intended for human use only, where, after disposal by consumers, they are processed along with their packaging. Pursuant to these regulations, drugstores and pharmacies, which are collection points for unused/expired drugs, are obliged to purchase, provide and maintain containers for the collection of unwanted pharmaceuticals in their facilities in a proportion of at least one fixed collection point for every ten thousand inhabitants [35]. However, it should be noted that these regulations apply only to medicines intended for humans. Moreover, only pharmacies located in cities of more than one hundred thousand inhabitants are obliged to participate in the above-mentioned system [36].

### 4.2. High Income Countries

In Kuwait, there are no guidelines for the acceptance and disposal of medicines returned from households. There is only a guideline issued by the Ministry of Health that pharmacies are required to return unwanted medications generated from their stock, for any reason such as expiry or overstock, to the Central Medical Stores [36]. Moreover, in Saudi Arabia, no official state guidelines have been issued by the Ministry of Health or protocols for the disposal of unwanted and unused medications or medical waste [37]. However, it is possible to return such drugs to a pharmacy [38]. A survey conducted by Alhomoud et al. (2021) [37] among undergraduate or postgraduate students of pharmacy indicated that pharmacies are the best place to return unused medications (67% of respondents). Moreover, according to respondents, the authorities responsible for the safe disposal of medications should be the Food and Drug Administration (90%) and pharmaceutical companies (87%), followed by pharmacies (81.5%) [37]. There are no regulations in Israel regarding the collection of pharmaceutical household waste. However, in 2001, the Ministry of Health published a circular that instructed health maintenance organization pharmacies to receive unused medications from any individual [39].

In the European Union, the obligation to implement appropriate collection systems of medicinal products that are unused or have expired results from Art. 127b, Directive 2004/27/EC of the European Parliament and of the Council of 31 March 2004, amending Directive 2001/83/EC on the Community code, relating to medicinal products for human use (OJ L 136, 30.4.2004, p. 34–57). In most European countries, the collection system includes either take-back to the pharmacy or drop-off at waste collection points, depending on the policy and strategy implemented in each country or region. Some pharmacies take part in the process on a voluntary basis, while some are obliged by law to participate in a take-back scheme. Croatia is an example of a country where pharmacies are obliged to accept unused/expired medicines from patients. By law, pharmacies are considered producers and holders of drugs and therefore are obliged (in accordance with the ‘polluter pays’ principle) both to receive drugs from patients and bear the costs associated with it [40]. Additionally in Serbia, pharmacies are obliged to offer unnecessary pharmaceuticals to patients and then transfer them to manufacturers, return them to wholesalers or transfer them to specialized companies for destruction [41].

In France, by Law No. 2007–248 of 26 February 2007, pharmacies are required to accept unwanted medications from patients [42]. The collection of unused medicinal drugs functions within Cyclamed, which is a nonprofit organization, in operation since 1993, in which dispensing pharmacists, wholesale distributors and drug companies are involved and whose purpose is to collect expired and nonexpired unused drugs that patients bring back to pharmacies for disposal [43]. In this system, wholesale distributors are responsible for delivering empty containers to pharmacies and picking up filled containers, while pharmacies are responsible for the free-of-charge collection of unused medicines from patients. Medical waste disposal is financed by Cyclamed through contributions from pharmaceutical companies [44]. Cyclamed succeeds in collecting 62% of unused medications [45].

The current legislation in Romania is designated by the National Public Health Institute as the authority responsible for approving on-site collection by the separation of waste accumulated from medical activities. However, the legislation covers only, for example, pharmacies, drug stores, distributors of medicines and pharmaceutical products, production units, the warehousing and storage of medicines and biological products or research institutes, while expired medicines derived from the population should be submitted to the nearest pharmacy or pharmaceutical point [46]. However, in the case of medicinal waste collected from the population, the law is not sufficiently explicit with regard to on what basis such waste is to be collected from patients and who will bear the costs of the disposal of medicines handed over to pharmacies: manufacturers, pharmacies or local authorities [47]. In a study concerning the opinions of pharmacists on the legislation and the procedure for collecting medical waste, just over 65% said they were dissatisfied with the current procedure, more than 40% of the investigated pharmacists considered current legislation to be incomplete and about 20% considered the law to be ambiguous, while almost 13% believed the law to be clear and easy to apply [47]. Furthermore, over 92% of respondents thought that the costs of the disposal of returned medicines should not be borne by pharmacies but by patients, local authorities, the Ministry of Health, the Ministry of the Environment and other entities (government, manufacturer or supplier, drug-issuing pharmacy, authorized ecological unit) [47]. At the same time, it should be noted that the regulations indicating that expired medicines derived from the population should be submitted to the nearest pharmacy or pharmaceutical point were introduced only in 2014 [46]. Poland is an example of a country that lacks clear legal provisions regarding the collection of unnecessary drugs from residents. On the one hand, municipalities are responsible for organizing a system of collecting municipal solid waste from residents; on the other hand, there are no precise regulations that would impose an obligation to segregate pharmaceutical waste [8]. Therefore, the system is based on the principle of the voluntary participation of entities such as pharmacies or health centers [48]. In Portugal, the disposal of household pharmaceutical waste is regulated by the National Environment Agency, but the national entity responsible for the collection and treatment of unused pharmaceutical products is Valormed, a nonprofit society created by the pharmaceutical sector (industry, distributors and pharmacies). It provides pharmacies with special boxes to collect pharmaceuticals returned by the public. Then, pharmaceutical waste is transported to sorting facilities, from where it may be sent for incineration [49]. In 2002, an integrated system for the collection and management of unused and expired drugs, called SIGRE (Sistema Integrado de Gestión y Recogida de Envases—Integrated Packaging Management and Collection System), was developed by the Spanish National Association of the Pharmaceutical Industry [50]. The pharmaceutical industry finances the work of SIGRE, which is responsible for the management of pharmaceutical waste and for the implementation of ecological campaigns [44]. The pharmacies with SIGRE collection points play a major role in the system [51]. However, wholesale distributors are responsible for the collection of pharmaceutical waste from pharmacies and its storage until disposal [44].

In the US, in 2007, the Food and Drug Administration and the White House Office developed and published, under the National Drug Control Policy, the first guidelines for consumers on drug disposal, which indicated that the proper way to dispose of drugs was to throw them into the garbage [52]. The Safe and Secure Drug Disposal Act, enacted in 2010 in the US, mandated the Drug Enforcement Administration (DEA) to develop and implement legislation that specifies methods for transferring unused or unwanted controlled pharmaceutical substances to authorized entities for disposal [53]. The DEA promulgated the Final Rule on the Disposal of Controlled Substances in 2014, which gives patients the opportunity to return unused drugs through a take-back event, dropping them at authorized points (for example, hospitals, pharmacies) or sending substances to authorized collectors in mail-back envelopes that meet the criteria of the DEA rule [54]. If, however, the drug take-back or collection program is not available, the guidelines issued by the Environmental Protection Agency (EPA) are to be followed. According to these guidelines, drugs should be removed from their original containers, placed into a disposable container or into a sealable bag, mixed with an undesirable substance such as cat litter or used coffee grounds and disposed of in the garbage [55].

## 5. Methods of Disposal of Unused/Expired Pharmaceuticals

**Figure 1:**
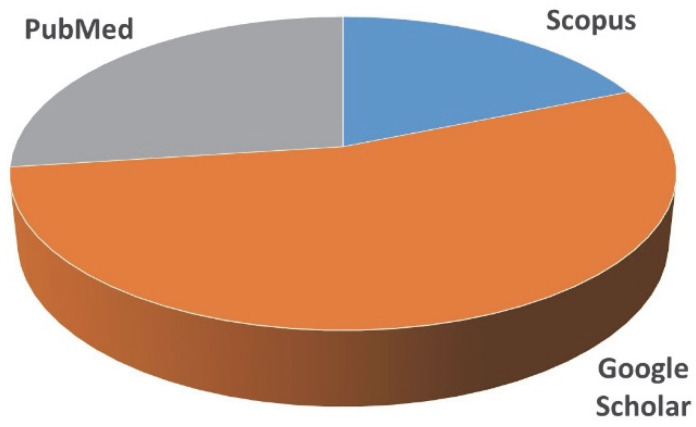
The number of articles from different sources.

**Figure 2:**
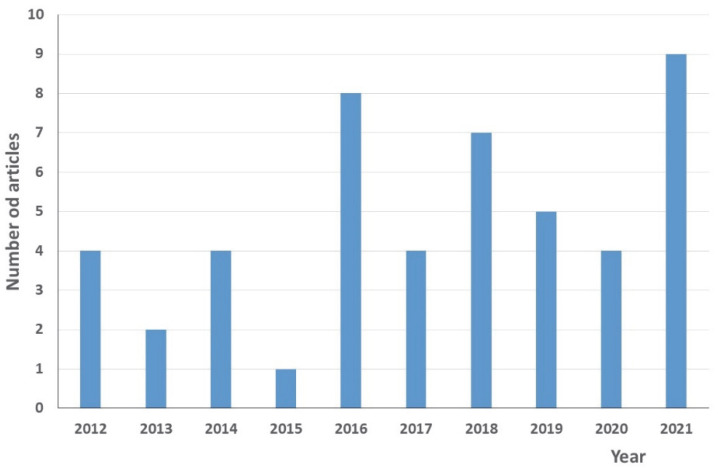
The number of articles in particular years.

In order to answer the question of how residents of various countries deal with unused/expired pharmaceuticals, 48 scientific articles published from 2012 to 2021 from different sources were analyzed (Figure 1 and Figure 2).

The analysis of literature shows that the main way to dispose of unwanted drugs is by throwing them into the garbage (Table 1). The inhabitants of both highly developed and low- and middle-income countries admit to this method of disposing of unwanted drugs. Indeed, in many countries, especially in Africa and Asia, throwing medications into the trash or pouring them down the sink or toilet is the only way to dispose of them (Table 1). Some respondents, especially from Tanzania, Uganda, Ghana, Indonesia, Malaysia and Saudi Arabia, give unused medications to family, acquaintances, friends or neighbors [24,26,28,37,56,57]. The reuse of unused pharmaceuticals is against the law in many countries, e.g., Poland or the UK, and taking such donated drugs may have negative effects on health, due to the conditions of their storage or the fact that they may be inappropriate [56,58]).

The reasons why respondents use improper methods of disposal are primarily:

− A lack of education or inadequate education in this field;
− A lack of an appropriate system or program for the return of unused drugs;
− Getting used to certain behaviors;
− Convenience;
− A lack of punishment (no responsibility).

In Tanzania, 91.4% of respondents were not aware of the existence of proper medicine disposal methods [26]. This is due to the fact that patients are not educated in this area or the methods of education are ineffective. For example, Yu et al. (2019) indicated that 79% of young adults and 88% of elderly people in China had not received advice on how to deal with unused medicines [67]. Similar results were obtained in studies conducted among residents of Saudi Arabia (73% and 80%, respectively) [76,77]. This problem also concerns specific categories of drugs, such as opioids. Among 300 adult cancer out-patients receiving opioids in Texas (USA), 223 (74%) were unaware of proper opioid disposal methods, and 138 (46%) had unused opioids at home [84].

On the other hand, most patients are aware that throwing unwanted medications into the trash or pouring them into the sewage system has a negative impact on the environment and health. For example, in Jordan, 72.5% of participants said they knew that the improper disposal of medications could harmfully affect the environment and health [72], and 0.86% of households in the US said that flushing unwanted medications down the toilet or sink may result in medications contaminating the water supply or negatively impacting the environment [52]. These results are comparable with the results obtained for the populations of Ethiopia, Malaysia, Afghanistan and Portugal [23,24,25,49,65].

Respondents indicate that it should be the responsibility of health care professionals and pharmacists to provide information on the handling of household pharmaceutical waste [72,85]. Providing appropriate guidance, in the opinion of respondents, could control or minimize medication wastage [25]. At the same time, people who receive instructions regarding proper disposal are more likely to return unused pharmaceuticals to a pharmacy [77]. This means that pharmacies play an important role in the system, not only as places for collecting unused pharmaceuticals but also in the field of environmental education. However, in order to provide adequate information, providers must have this knowledge themselves. A survey conducted in the Kingdom of Saudi Arabia among undergraduate or postgraduate pharmacy students indicated that more than half (60%) had never received any information during their studies or training on how to store or dispose of medications. Moreover, 89% reported previously disposing of unused medicines mainly in household garbage [37]. On the other hand, between 73.3 and 75.3% (depending on the dosage form) of community pharmacists in Saudi Arabia returned unused drugs to the pharmaceutical distributors, and only between 1.1 and 4.4% poured them down the sink or toilet [38]. These results are similar to the results obtained in Nigeria. A study conducted among community pharmacies in Anambra State in Nigeria indicated that the most common method of drug disposal was via the National Agency for Food and Drug Administration and Control or drug distributors (above 60%). However, about 20% of drug disposal was via rubbish bins [63]. In contrast, most pharmacists in Kuwait disposed of pharmaceuticals by throwing them in the trash (73%) or pouring them down the toilet or sink (9% and 32%, respectively). At the same time, over 80% of these pharmacists are aware that this method of disposing of pharmaceuticals can cause damage to the environment [36]. A phone and online survey by Bungau et al. (2018) carried out on a group of 521 pharmacists from Romania indicated that 53.6% consider themselves sufficiently informed about the waste disposal legislation, only 19.3% consider themselves highly informed and almost 9% believe they are little or not at all informed [47]. Only 15.9% of pharmacists from California (US) correctly selected all of the appropriate methods of medication disposal that could be recommended to patients in their communities for non-controlled substances, and only 10.1% were able to recognize all of the appropriate methods of disposal for controlled substance medications [86].

Accordingly, it is important to properly educate pharmacy students, pharmacists and health care staff in this field. In the aforementioned survey, Alhomond et al. (2021) showed that among the students who received information on the appropriate disposal of medicines, 85% did not dispose of unused or expired medications in household garbage (while 15% did), and of those who had never received such information, 91% did (9% did not) dispose of medications in household garbage [37]. Of the people in Ireland who obtained advice from a health care professional regarding disposal, 75% chose an appropriate method compared with 25% who did not [81]. Tabash et al. (2016), in their research, assessed the impact of an educational program on the knowledge, attitude and practice of health care staff regarding pharmaceutical waste management. The survey was carried out among various health care staff working in five governmental hospitals in Gaza before, after and six months following the implementation of the educational program. It was found that the educational program led to a significant improvement in the knowledge, attitude and practice of health care staff regarding pharmaceutical waste management. Knowledge and practice levels increased from 50 to 75% [87]. In addition to information obtained from pharmacists, doctors and other health care professionals, patients obtain knowledge about the appropriate disposal methods of unused drugs from books, the media, the Internet, family members and friends. Yu et al. (2019) noticed that for young adults and aged people, family members such as parents and children appeared to be the main advisors on the disposal of medications [67]. In a different study, 54% of respondents in the US reported having looked for drug disposal information, with the Internet being the primary source of information [6], whereas 56.1% of patients from Jordan reported that social media was the preferred method of education regarding the disposal of unused or expired medications [72]. Electronic media are, according to respondents among the Ethiopian community, the best source of ecological information (49.6%), with doctors also a good source (24.5%), whereas, only 8.5% of the participants in this study indicated pharmacists [23].

One source of information is educational campaigns and programs carried out by national and local governmental bodies as well as environmental organizations and private institutions. Public education campaigns on pharmaceutical waste management in the Bihor county (Romania) population resulted in a significant increase, from 1.1% to 87.3% in 6 months, in the number of patients who returned expired and/or unused drugs to pharmacies [46]. The implementation of an educational program in a private communication company in Turkey contributing to the proper storage, use and disposal of pharmaceuticals resulted in a positive change in the drug disposal behavior of 46.5% of the employees who participated in the program [80]. In Spain, one of the components of the SIGRE system, beyond receiving leftover medication from patients, is educational campaigns. For example, in 2018, a campaign under the slogan “Thanks for lending us a hand” was launched. The goal of this campaign was a more responsible use of drugs and encouraging the return of unwanted drugs, especially antibiotics, to SIGRE collection points located in pharmacies [51].

The International Pharmaceutical Federation (FIP), in 2015, prepared the document: “Green pharmacy practice—Taking responsibility for the environmental impact of medicines”, in order to provide pharmacists’ associations with useful information in this field. This reference document proposes solutions for developing green pharmacy policies, such as: creating an adequate legal framework at the national level, establishing effective pharmaceutical waste disposal practices, organizing continuous training programs for pharmacists and other health professionals and running informational and educational campaigns for patients in order to reduce and properly dispose of pharmaceutical waste [88].

In Australia, in 1998, the Return Unwanted Medicines (RUM) project was introduced by the government. This program provides the free option for the public to return unwanted and expired medicines to community pharmacies [89,90]. Returned medicines are deposited in bins situated within any community pharmacy, which are then collected by pharmaceutical wholesalers, transported to registered incineration sites and disposed of by high-temperature incineration [89,91]. Although in the years 2000–2018, there was an increase in the amount of unwanted medicines collected in pharmacies, from 19.6 to 66.4% per month, a 2016 survey of 4302 Australian adults found that only 17.6% of them had heard of the RUM project [83,92].

A return or take-back voluntary program was also implemented in Malaysia. Under this national program, patients can return unused drugs to pharmacies, hospitals and health clinics that are subordinate to the Ministry of Health Malaysia [31]. Although this program has functioned in Malaysia since 2010, the analysis of patient behavior indicates that still, the main method of disposing of unwanted drugs is by discarding them in the trash (Table 1).

Canada is one of the countries that started introducing unused drug return programs by consumers in the 1990s. In 1996, the EnviRX program introduced in British Columbia was aimed at the possibility of returning unused/expired drugs by patients [92]. Residents can also return unused/expired prescription or OTC drugs in Canada for free. Due to the fact that Canada consists of various provinces and territories, there is a mixture of guidelines, regulations and programs for the appropriate disposal of pharmaceuticals [42]. For example, in British Columbia, Manitoba, Ontario and Prince Edward Island, the Medications Return Program was implemented. This program is operated by the Health Products Stewardship Association (HPSA). The HPSA represents producers of health products in Canada, and it is financed by them [93]. As part of the program, since the beginning of its operation, 3,783,069 kg of medications have been collected [92]. Alberta, Saskatchewan and Nova Scotia have voluntary programs, administered and monitored by their respective pharmacy associations [42]. The effect of the take back programs introduced in the 1990s is the large (compared and other analyzed countries) participation of patients in the system of drug return to pharmacies. A survey conducted by the Canadian statistical office as part of the Canadian Environmental Sustainability Indicators program in 2011 showed that over 63% of citizens return medicines to the supplier, retailer, pharmacy or doctor, while 21% throw them into the garbage, and 5% pour them into the sink or toilet [94]. In September of 2010, the US Drug Enforcement Administration (DEA) established a nationwide program called the National Take-Back Initiative [95]. As part of the program, a biannual series of National Prescription Drug Take-Back Days are organized [96]. The aim of the program is to provide the opportunity to donate unwanted drugs and educate the public about not abusing drugs and about the proper way to dispose of them. During Take-Back Days, collection points for unwanted drugs are organized in cities throughout the United States. For example, during April 2021, the 20th National Take-Back Days managed to collect 420 tons of unwanted drugs at 5060 points across the country [97].

As reasons for the inappropriate disposal of unused drugs, residents also indicate convenience or habit. Patients from Portugal indicated that in order to increase the amount of medications returned to pharmacies, it would be important for the number of collection points to be increased, thus reducing the distance to them [49]. More than half of the respondents who declared their willingness to participate in Take-Back Days in Texas (US) admitted that they would not take part in this initiative if they had to travel more than 5 miles to do so [95]. On the other hand, among the survey participants in Saudi Arabia who were unwilling to properly dispose of medications, above 50% said they had no specific reason for this [37].

Some researchers have reported that sociodemographic factors such as education, income level, household size and gender influence a household’s environmental behavior [98,99,100]. The analysis of literature reports shows that in some studies, such relationships occur, while in others, age, gender and education do not affect the method of disposal of unwanted drugs. Shaaban et al. (2018) and Hassali and Shakeel (2020) indicated that highly educated respondents are more likely to return medications to a pharmacy and are more likely to be cognizant of the detrimental consequences of inappropriate waste disposal than those of lower educational levels [25,77]. The analysis of the behavior of pharmacy students in Saudi Arabia showed that the percentage of female students found to throw unused or expired drugs into the household garbage was significantly higher compared with the male students [37]. On the other hand, Akkici et al. (2018), in studies conducted among respondents in Turkey, observed that a higher percentage of women declared having changed their unused drug handling practice (49.9%) than men (38.5%) [80], whereas age, gender or education did not affect the environmental behavior of the respondents in Romania, the USA, Thailand or Ireland with regard to the handling of unused drugs [6,46,59,71,79].

An important issue that should be given attention is keeping unused medicines at home for various reasons. In Tanzania, the majority of households (96%) kept unused or expired medications [26]. More than 85% of respondents in Indonesia reported storing unused medications in their homes [28]. In Malaysia, 70.6% of respondents claimed to have some bought medications that remained unused in their homes [25]. Almost all residents of Ghana (98%), in a study by Abruquah et al. (2014), declared that they had unwanted medications in the home that they wished to dispose of [57]. Keeping unused or expired drugs is not something that concerns residents of developing countries. According to the studies of Wieczorkiewicz et al. (2013), a quarter of respondents had expired drugs, both prescription and nonprescription, in their households. In one study, 61.8% of the residents of Cook County (Illinois, USA) indicated that the longest time that they had ever stored expired medications was less than 1 year, and 2% stated that they had stored expired medications for longer than 10 years [52]. Moreover, Vatovec et al. (2017) showed that drug expiration was not the main reason for disposing of medications (1% prescription, 8% OTC) [29]. In the case of households in Serbia, drugs that were no longer used were still kept by about 95% of residents [41]. Unused medicines were kept in the home by 88% of respondents in Ireland [81], and 44.4% of Serbian households indicated that they had expired medications [82]. Residents of Cyprus also indicated that they kept quantities of unused drugs remaining after their medical treatment (76.8%) [9]. At the same time, some studies indicate that elderly people rather than younger people prefer to keep unused medicines at home, and more women kept unused drugs at home than men [67,80].

Unused pharmaceuticals were kept at home for several reasons. The first is the belief that they may be useful in the future. This was the main reason for residents of Malaysia (70.4%), Portugal (43%), Cyprus (93.4%), Uganda (55.4%) and Ireland (57%) to keep unused medicines at home [9,24,49,56,81]. Another reason is that the drugs have not yet expired. For instance, 33% of Portuguese residents used this reason to keep unused drugs, even if they assumed that they would not use these drugs in the future [49]. Massoud et al. (2016) indicated that 67% of residents of the Beirut area disposed of unused drugs due to the expiration of the medication [73]. Moreover, a study in Malaysia showed that drugs are disposed of after not being stored correctly and medicines turned bad (74.4%) or when medicines smelled bad, tasted bad or looked bad (72.9%) [24]. Other reasons residents keep unused medications include: giving to friends or someone in your family who may use them [49,56] or fear that they will not be able to find the same drug on the market [9]. A further reason that people keep unused/expired medicines at home is that they are unsure of how to dispose of them. More than half of the respondents indicated this reason in research conducted by Azad et al. (2012), Yu et al. (2019) and Ong et al. (2019) [24,67,69].

## 6. Environmental Impacts of Improper Disposal of Unused/Expired Pharmaceuticals

Pouring unwanted drugs into toilets or sinks causes them to be transported through the municipal sewage system to municipal wastewater treatment plants. Wastewater treatment plants, both in undeveloped countries (if they exist) and in developed countries, focus mainly on treating wastewater with regard to nitrogen and phosphorus compounds or suspended solids. In the European Union countries, there are no regulations obliging member states to use technologies that enable the removal of micropollutants, including pharmaceuticals, from wastewater. According to Council Directive 91/271/EEC of 21 May 1991 concerning urban wastewater treatment (OJ L 135 30.5.1991, p. 40), member states shall ensure that the treated wastewater released into water meets the requirements for biochemical and chemical oxygen demand and total suspended solids, and in the case of emissions, it is sensitive to specific nitrogen and phosphorus contents. In effect, micropollutants, including pharmaceuticals and their metabolites, end up in water reservoirs, such as seas or rivers, which may pose a threat to organisms living in them. For example, according to information contained in the UNESCO/HELCOM report, the main pathway of pharmaceuticals into the freshwater and marine environment in the area of the Baltic Sea is via the discharges of municipal wastewater treatment plant effluents [16].

The disposal of pharmaceuticals in conventional wastewater treatment plants using mechanical, biological and chemical processes depends on the two processes of sorption and biodegradation [101]. For example, inter alia, carbamazepine, diclofenac, metoprolol, tramadol and rosuvastatin display low sorption on sludge [102]. In effect, these compounds may pass into the liquid phase. Therefore, only about 30% of diclofenac is removed, while for example paracetamol is removed up to 100% [103]. In addition, the degradation process may result in higher concentrations of some compounds, such as carbamazepine, being observed in the effluent than in the influent [104]. Moreover, compounds present in wastewater can undergo degradation processes and react with other compounds in the environment. As a result, compounds with higher toxicity than the original compounds can be formed [105]. An even greater environmental problem occurs when there are no sewage systems and no wastewater treatment systems. Large portions of African communities do not even have proper sanitation systems and modern ablution facilities that can be flushed with fresh water to direct human waste into WTP [106,107]. As a result, pharmaceuticals and their metabolites enter directly into water and soil. The threat to water (including groundwater) and soil also results from throwing unused/expired pharmaceuticals into household rubbish bins or directly into the environment. Household waste is disposed of in landfills. In a study by Lu et al. (2016), 15 compounds, among others, atenolol, ibuprofen, ketoprofen, diclofenac, gemfibrozil, benzophenone, carbamazepine erythromycin, amphetamine, methamphetamine, ketamine, ephedrine, flunitrazepam and codeine were found in leachate from municipal landfills [108]. Carbamazepine and primidone were detected in all tested leachate samples from five landfills in the USA [109].

Pharmaceuticals are designed to elicit a biological response in organisms even at low concentrations. Accordingly, those in the aquatic environment might also produce a biological response in nonspecific organisms [110]. Many of these compounds are toxic to aquatic organisms. For example, acetaminophen exhibits low toxicity to the bacteria *Allivibrio fischeri*, whereas its toxicity to *Daphnia magna* is high [111,112]. Moreover, metoprolol exhibited a negative chronotropic effect on the heart of *Daphnia magna* at a high concentration exposure, and, among analgesics, the most toxic effect was reported of EC_50_ below 100 mg L^−1^, while phytoplankton is highly sensitive to metoprolol in acute and high-level exposure with EC_50_ of 14.5 mg L^−1^ at 96 h post exposure. Diclofenac also causes gill alterations in trout fish [113]. In addition, it should be noted that the ecotoxicological effects of pharmaceuticals are still unknown [114], e.g., 88% of human pharmaceuticals do not have comprehensive environmental toxicity data [115]. At the same time, pharmaceuticals and their transformation products are present in a mixture, and therefore it is difficult to clearly define their negative impacts on organisms.

In recent years especially, the problem related to the release of antibiotics into the environment has intensified, causing the development of antibiotic-resistant bacteria and the environmental occurrence of antibiotic-resistant genes [116]. This phenomenon results in resistant bacteria that are able to survive in the presence of an antimicrobial in concentration that is usually sufficient to inhibit or kill microorganisms of the same species [117]. The human health risk of ARGs is due to the fact that antibiotic resistance can be transferred from environmental components to drinking water sources [118]. In Europe, 75% of inhabitants use groundwater as their source of potable water [119]. At the same time, both in Europe and in other parts of the world, pharmaceuticals are detected both in groundwater and drinking water. For example, in groundwater samples in Barcelona, the most frequently detected compounds were antibiotics, which were present at concentrations reaching 1000 ng L^−1^ [120]. On the other hand, analyses of tap water samples taken in Northern Kunming (China) showed that out of 17 examined antibiotics, 9 antibiotics, inter alia, trimethoprim, sulfamethoxazole, oxytetracycline, metronidazole, dimetridazole, azithromycin, clarithromycin and roxithromycin were detected in over 80% of all samples. The average concentration of total antibiotics in tap water was 10 ng L^−1^ [121].

Accordingly, in 2015, the WHO adopted a global action plan on antimicrobial resistance, including antibiotic resistance. The aim of the plan was, among others, to increase the knowledge and awareness of antimicrobial resistance and to conduct activities related to optimizing the consumption of antibiotics [122]. Moreover, the European Union, in the EC Communication from 29 June 2017, announced the European One Health Action Plan against Antimicrobial Resistance [17].

At the same time, it should be noted that the COVID-19 pandemic resulted in an increase in the consumption of certain drugs, including antibiotics, and thus an increase in their amounts in the sewage stream [123]. Due to the lack of drugs that were dedicated to the fight against the SARS-CoV-2 virus, drugs from various therapeutic groups were tested, i.e., antiviral drugs used in AIDS, antimalarial drugs (e.g., chloroquine and hydroxychloroquine), antibiotics (e.g., azithromycin), painkillers and combinations of these drugs (e.g., hydroxychloroquine and azithromycin) [124]. Literature research by Morales-Paredes et al. (2022) indicated that the concentrations of most drugs used in COVID-19 therapy in the aquatic environment increased during the pandemic (e.g., azithromycin concentrations in surface waters from 4.3 ng L^−1^ before the pandemic to 935 ng L^−1^ during the pandemic) [125].

## 7. Recommendations

Reducing the amount of drugs in the environment, and thus their negative impact, is a multistage process and includes, inter alia, the rational prescription of drugs, the limitation of drug consumption, legitimate self-medication and proper disposal. Social awareness of the problem plays a key role in each stage of the process.

### 7.1. Prescribing and Purchasing Pharmaceuticals

Prescribing excessive amounts of drugs increases their consumption and storage. Storing medicines at home and their consumption by a person other than the person for whom they are prescribed can increase the risk of adverse effects. The accidental intake of drugs by children may be a problem. In addition, the storage of drugs means that consumers will, most likely, eventually dispose of them when they have expired. In a survey conducted by Naser et al. (2020), Jordanian respondents indicated that from their perspective, the best way to reduce drug waste was by dispensing medications only as required (42.4%), prescribing medications rationally (35.4%) and giving proper advice to consumers (33.5%) [72]. Similar conclusions were obtained in the research by Hassali and Shakeel (2020). Respondents from Malaysia suggested that the provision of appropriate directions by health care professionals (73.2%) and prescribing medications in quantities for a duration that ensures patient adherence (26.7%) could control or minimize medication wastage [25]. Therefore, an important role in the process of limiting the amount of drugs in the environment is played by health care workers authorized to prescribe drugs and pharmacists who advise patients to buy OTCs. At the same time, pharmacists should also be educated because they interact greatly with patients and can change inappropriate consumer habits. This is especially important in the case of the currently very common problem of self-medication. The effect of self-medication is not only the purchase of large amounts of drugs by patients, their subsequent storage and then their disposal but also negative consequences for the health of patients resulting from, for example, interactions between drugs or the risk of side effects. Despite the fact that the regulations for the promotion of pharmaceuticals, especially in the EU, point to the importance of monitoring drug advertisements to ensure compliance with laws and regulations and using adequate sanctions to prevent violations, the legislation consists of bans on public advertisements of prescription-only drugs. However, the legal rules still do not reduce the patients’ needs, and raising social awareness is a key point for a sustainable pharmacy market.

The COVID-19 pandemic has resulted in an increase in demand for the purchase and storage of drugs, especially those for chronic diseases. The phenomenon of “panic buying” was particularly visible in richer geographical areas [126,127]. Elek et al. (2021) reported that in Hungary, the duration of therapy with regard to pharmaceutical purchases increased by more than 30% in the month when major lockdown measures were announced, and this refers to almost all categories of pharmaceuticals [127]. Lockdowns and restricted access to doctors were also associated with an increase in the problem of self-medication among patients.

### 7.2. Disposal of Pharmaceutical Products

There is no doubt that the ways in which society wrongly disposes of unused/expired drugs have a negative impact on the environment. Accordingly, safe drug return systems and programs need to be established and developed. Pharmacies play an important role in such systems as the points of return of pharmaceuticals, due to their general availability and number. In some countries, they are obliged to be included in the system of collecting unused/expired medicines from households (e.g., Croatia, France), whereas in others, they operate on a voluntary basis (e.g., Poland). The next stage is to educate the public on the proper handling of unwanted drugs, including collection systems and drug take-back programs. For example, although the RUM project in Australia was launched in 1998, a survey conducted in 2016 showed that less than 18% of Australian respondents had heard of it. On the other hand, 91.7% of respondents who were previously unaware of the scheme stated they would now use it [90]. At the same time, attention should be paid to adjusting educational tools to the target group and national specificity. In the case of older people, in our opinion, the participation of pharmacists in the education process will be important, while in the case of young people, electronic media and social networks may prove effective.

The COVID-19 pandemic and blockades, and thus limits on the possibility of movement in different countries and patients’ fear of getting sick, could reduce the willingness and actions of patients in the pharmaceutical waste collection system [128]. In the USA, Drug Take Back Day, which was planned for April 2020, was canceled due to the pandemic [129].

Pharmacy reuse (sometimes referred to as recycling) is a complex issue, facing concern, especially regarding safety and liability. The controlled reuse of drugs can be a way to reduce their negative environmental impact. Reuse can also help reduce the accumulation of drugs in society. However, the problems that appear in this case arise from the storage methods of medications in households, including poor storage conditions such as humidity and temperature, which are not regulated. Research conducted in the Netherlands and Great Britain showed that over 50% of respondents were willing to use medications returned unused to the pharmacy by another patient as long as the quality of these medicines was verified [130,131]. In addition, an experiment by Lam et al. (2021) in Great Britain showed that the integration of sensors that measure and track the interaction of the storage conditions (e.g., temperature, light, humidity) on drug packaging, and the guarantee that the quality of drugs will be visually assessed by the pharmacist, meant that the respondents would be more willing to participate in a system for the reuse of medications [58].

Despite these repository programs, the collection and redistribution of unadulterated prescription drugs are not allowed by dispensing laws in EU countries. The reuse of still-usable drugs in modern, tamper-evident packaging is still dangerous with regard to unknown storage conditions.

### 7.3. Waste Management and Wastewater Treatment

Because the main methods of disposing of unused/expired drugs by respondents from various countries include throwing them into the garbage and pouring them into the sink/toilet, waste management and wastewater treatment from pharmaceutical residues should be developed at the same time. On the one hand, some countries (e.g., Switzerland, Germany) started introducing additional methods of wastewater treatment (e.g., ozone, granulated activated carbon) in wastewater treatment plants to remove micro-pollutants such as pharmaceuticals and their metabolites. In other countries, however, wastewater treatment systems are completely lacking. For example, in Ghana and India, only 7.9% and 30.7%, respectively, of the wastewaters are treated, which results in the presence of pharmaceutical residues in the aquatic environment [103]. This same situation occurs in the case of household waste. Incineration is the main method of waste management in some countries, such as Germany and Sweden, and in other countries such as Malaysia, Poland and Romania, landfilling is the leading practice in the management of solid waste [24].

An important area in the field of wastewater treatment seems to be the development of new, more effective and low-cost methods of the elimination of pharmaceutical residues from wastewaters. Among the treatment methods, photocatalytic methods play a special role and have been recognized as one of the most promising methods for the elimination of organic pollutants in environmental matrices [132]. Although TiO_2_ is the most frequently used photocatalyst, other such semiconductors show good elimination of organic pollutants. For example, meso-tetra (4-carboxyphenyl) porphyrin (TCPP)/Bi_12_O_17_Cl_2_ shows photodegradation efficiency of 79.4% for tetracycline [133]. In the case of tetracycline, an effective level of degradation can be obtained by also using the combination of Cd_0.5_Zn_0.5_S nanoparticles and Bi_2_WO_6_ microspheres [134].

## 8. Limitations of the Study

This study had some limitations. The selected period (2012–2021) from which the publications were analyzed allows data to be shown only from selected countries. Published studies from particular countries may not be representative of the entire population of the country because they are conducted, for example, on a specific, selected group of respondents, such as students or residents of large cities. In addition, some research was conducted using online surveys, which may also affect the results (e.g., the tendency to behave differently on the Internet compared with the real world, access to the survey only for people who have and use IT tools).

## 9. Conclusions

The conducted review showed that the basic way for patients in different countries to deal with unused/expired drugs is to throw them into the garbage and/or pour them down the sink. This happens even despite the fact that many countries have systems for collecting unnecessary medications from patients. In view of the above, investigating the reasons why expired/unused pharmaceutical products become waste plays a key role in reducing the problem. There is a need for more research to explore this problem. It seems that a global system based on the obligatory collection of unused/expired medicines from households is needed. Simultaneously, with the development of this system, it is necessary to effectively educate societies about the impacts of improper drug disposal on the environment. It is equally important to conduct research on developing new or modifying existing methods of wastewater treatment from hazardous compounds, especially now that the problem of microbial resistance is becoming more and more serious.

## Figures and Tables

**Table 1:** The way of dealing with unused/expired medicines by respondents around the world.

Region	Country	State or City	Years of Research	Type of Respondents	The Method of Conducting the Research	Research Group	Procedure Dealing with Unused/Expired Drugs by Respondents				Reference
							Return to Pharmacy/Pharmaceutical Distributor/Health Facility/to Drug Take-Back Program [%]	Throwing in the Garbage [%]	Toilet or Sink Discharge [%]	Other [%]	
North America	USA	Cook County, Illinois	2009	Household members over the age of 18	Phone interview	445	16.0	59.0	31.0	6.0—give to someone else	[52]
		Southern California	2011	Campus community	Web-based survey	238	11.2—pharmacy 1.8—physician’s office	62.7	18—toilet 4.3—sink	8.0	[59]
		Vermont	2014	University students from Burlington	An online survey	358	2.0	25.0	1.0	-	[29]
		Vermont	2016	Vermont residents over the age of 18	Telephone polling	252	37.0	32.0	12.0	-	[6]
South America	Brazil	Lages	2018	Residents of two districts of Lages over the age of 18 who had a fixed residence	Face-to-face interviews	309	15.3 (unused) 8.7 (expired)	39.6 (unused) 69.6 (expired)	5.0 (unused) 11.1 (expired)	1.0—burn 8.9—give to someone else 30.2—keep at home (unused) 2.8—burn 0.5—give to someone else 4.6—keep at home (expired)	[60]
Africa	Ghana	Ashanti region	2012–2013	Residents of Konongo–Odumasi over the age of 18	Literate and semi-literate participants filled out the questionnaires themselves, while illiterate respondents were taken through structured personal interviews in the local dialect.	500	1.0	29.0	4.0	38.0— bury 21.0—give to someone else 7.0—burn	[57]
	Ghana	The Greater Accra Region	2014	One resident from each household, from Ga South Municipal Assembly over the age of 18	Face-to-face interviews	600	-	80.2	-	18.8—burn/bury/others	[61]
	Ghana	All country	no data	Residents of Ghana over the age of 18	The respondents answered the questionnaires either by themselves or with the assistance of the researcher. Some of the respondents needed assistance because they could neither read nor write.	no date	less than 4	more than 75	-	-	[22]
	Ethiopia	Harar city (eastern Ethiopia)	2018	One resident from each household over the age of 18	Face-to-face interviews	694	1.0	53.2	23.9	2.2—burn 1.9—give to someone else 1.9—donate to hospital 16.0—keep at home until expired	[23]
	Kenya	Nairobi City	No date	Populations of two distinctive groups of households: within the formal settlement (middle-class) and informal settlement (lower-class) areas	Face-to-face interviews	164	4.9—take-back programs 17.7—special garbage bins	28.7	25.0	9.8—burn 14.0—bury	[62]
	Nigeria	Anambra State (southeast Nigeria)	2016–2017	103 community pharmacies in the three senatorial zones in the state	Questionnaire survey and key informant interview. The questionnaire was distributed in community pharmacies. Pharmacists received, filled out and returned their questionnaires.	77	24.7–34.1 *—pharmaceutical distributors 29.6–36.1 *—the National Agency for Food and Drug Administration and Control bin	9.6–23.9 *	1.2–7.1 *	4.7–9.6 *—burn	[63]
	Tanzania	Mwanza City	2015	One resident from each household over the age of 18	Face-to-face interviews	359	-	59.1	12.5	8.4—burn 0.3—bury 17.9—give to someone else	[26]
	Uganda	Northern Uganda (four districts including Gulu, Nwoya (Acholi sub-region), Lira and Dokolo (Lango sub-region)	2012	One resident from each household over the age of 18	Face-to-face interviews	260	0.8	10.8	-	33.0—give to someone else 55.4—keep for future use	[56]
	Zambia	Lusaka	2019	Students from three higher learning institution	Face-to-face interviews	385	4.4	60	33.3	2.3—burn/bury	[64]
Asia/ East, South Asia	Afghanistan	Kabul	2016	Residents of Kabul over the age of 18	Face-to-face interviews	301	21.3 (unused) 7.3 (expired)	14.3 (unused) 77.7 (expire)	1.3 (unused) 12.0 (expire)	1.3—give to someone else 9.6—donate to hospital 52.2—keep at home until expired (unused) 1.3—give to someone else (expired)	[65]
	China	New Territories, Hong Kong Island, Kowloon Peninsular	2015–2016	Respondents from seven sites in the Hong Kong Special Administrative Region	In-street questionnaire surveys	1837	0.9	53.9	4.1—toilet 1.3—sink	0.5—burn 3.1—give to someone else 0.6—sell to others	[66]
	China	Wuhan	2018	Students from Wuhan University of Science and Technology	The participants completed the questionnaire themselves.	365	0.6	84.0	-	-	[67]
				Residents in retirement homes	The participants provided their responses marked by the researchers.	206	5.3	94.0	-	-	
	India	North India	No date	Dental students from second, third and fourth academic years aged between 18–25 years	Descriptive cross-sectional survey based on a structured questionnaire format	236	3.0	94.0	12.0—toilet 32.0—sink	-	[68]
	India	Haryana State	2018–2019	Students from the faculty of science of the university and college (a literate group)	An online survey	196	22.0	48.0	-	14.6—keep for future use	[32]
				Residents of nearby villages between 18 and 50 years (an illiterate group)	The participants were assisted in filling out the questionnaires offline.	491		85.0	-	32.9—keep for future use	
	Indonesia	Bandung (West Java Province)	2017–2018	Residents over the age of 18	Interviews	497	0.2 (expired)	29.0 (unused) 82.1 (expire)	1.8 (unused) 5.3 (expire)	4.4—give to someone else 0.4—donate to the hospital (unused) 4.0—burn 0.4—give to someone else (expired)	[27]
	Indonesia	Yogyakarta Province (three districts: Sleman, Bantul and city of Yogyakarta)	2018	One resident from each household over the age of 18	Interviews	324	3.1	71.6	17.3	23.2—give to someone else	[28]
	Malaysia	Selangor, Pahang	2011	Patients of the health center (Gombak campus, Selangor) and medical college of the International Islamic University Malaysia (Kuantan campus, Pahang), over the age of 18	The questionnaire was handed out in person, and the question sheet was collected with their answers.	885	6.0 (liquid) 8.0 (solid) 12.0 (ointments and creams)	27.0 (liquid) 65.0 (solid) 83.0 (ointments and creams)	62.0 (liquid) 1.0 (ointments and creams)	-	[69]
	Malaysia	Selangor	2018	Households in a residential area in Hulu Langat	Face-to-face interviews	103	25.2	63.1	2.9—toilet 8.8—sink	3.8—burn 3.8—bury	[31]
	Malaysia	Selangor	2019	Residents of Selangor over the age of 18	Face-to-face interviews with people in public places	426	8.2 (unused) 1.1 (expired)	47.4 (unused) 84.9 (expire)	5.8 (unused) 12.4 (expire)	7.2—donate to hospital/charity 11.5—give to someone else 16.1—keep at home until expired (unused)	[25]
	Malaysia	All country	2019	Residents of Malaysia over the age of 18	The participants could choose to use a web-based (Google Forms) or a paper-based survey (all potential public places across Malaysia).	483	17.0 (liquid) 28.0 (solid) 13.3 (ointments and creams)—return to pharmacy 15.5 (liquid) 17.6 (solid) 12.8 (ointments and creams)—dispose in biomedical waste bin	54.7 (liquid) 76.0 (solid) 61.1 (ointments and creams)	20.7 (liquid) 8.5 (solid) 2.5 (ointments and creams)—toilet 42.9 (liquid) 2.1 (solid) 3.7 (ointments and creams)—sink	15.7 (liquid) 31.5 (solid) 22.8 (ointments and creams) -give to someone else 3.7 (liquid) 11.4 (solid) 4.6 (ointments and creams)—burn	[24]
	Nepal	Lalitpur	2020	Undergraduate medical and dental students	An online survey	441	9.3	38.5	4.1	3.4-burn	[70]
	Thailand	Khon Kaen City (Ban Ped subdistri)	2009–2010	The survey was conducted by Thai villagers	Face-to-face interviews	311	1.0 (solid)	64.6 (liquid) 81.4 (solid) 66.6 (ointments and creams)	7.4 (liquid)	1.6 (solid) 0.6 (ointments and creams)—bury	[71]
Asia/Western Asia/ Middle East	Cyprus	District of Nicosia	no date	Residents over the age of 18	Convenience sampling	184	-	92.4	24.5	0.5—burn 8.2—give to someone else	[9]
	Israel	All country	2014–2015	Residents over the age of 20	Poll by the Central Bureau of Statistics	7000	10.7–18.6 (depends on region)	more than 85	-	-	[39]
	Jordan	All country	2019	Residents over the age of 18	The questionnaire was filled out by the patient or was completed with assistance from the pharmacist.	1092	11.8	62.4	9.3—toilet 12.0—sink	7.6—give to someone else 1.8—others	[72]
	Kuwait	All country	2009	Pharmacists working in the six government hospitals and in the polyclinics	The questionnaire was handed out in person. The authors waited until the participants filled out the questionnaire and handed it back.	144	10.0—return to Ministry’s Central Drug Store 6.0—disposed with hospital medical waste	73.0	9.0—toilet 32.0- sink	20.0—give to someone else	[36]
	Lebanon	Beirut Area	2014	Residents of 13 zones of Administrative Beirut Area	The questionnaires were distributed to selected houses in each residential zone. Only residential buildings (apartments and standalone houses) were included in the random sample selection process; commercial buildings were excluded from the study sample.	300	1.5 (liquid) 3.6 (solid) 0.7 (ointments and creams)	72.6 (liquid) 78.3 (solid) 86.9 (ointments and creams)	17.0 (liquid) 6.0 (solid) 5.5 (ointments and creams)	4.4 (liquid) 8.5 (solid) 2.5 (ointments and creams)—give to someone else 4.4 (liquid) 3.6 (solid) 4.4 ointments and creams)—other	[73]
	Palestine	Nablus City (North of Palestine)	2011	Patients of several primary and secondary care institutions as well as local community pharmacies	The survey was distributed to patients and pharmacists.	250	13.6	66.4	10.8	-	[74]
	Saudi Arabia	Riyadh City	2015	Patients and personnel of King Khalid University Hospital and King Saud University	The questionnaire was handed out in person. The respondents filled out the questionnaire and returned it.	1057	1.7—return to physician 1.7—return it to pharmacy 3.7—hazardous waste collection	79.2	7.0	1.6—give to someone else 0.6—other	[75]
	Saudi Arabia	Jeddah City	no date	Patients, family members and working staff of King Abdulaziz over the age of 16	The survey was distributed in person.	1171	13.6	72.8	4.6	2.6—give to someone else 1.1—burn	[76]
	Saudi Arabia	All country	2017	Residents over the age of 18	The questionnaire was sent to respondents across the country.	767	1.4—physician 6.5—pharmacy	62.9	16.6	9.1—give to someone else	[77]
	Saudi Arabia	Qassim Province	2017	Residents of Qassim Province over the age of 18	An observational cross-sectional survey	302	5.0 (unused)—pharmacy 5.0 (expired)—medical store	75.8 (expire)	28.5 (unused) 9.0 (expire)	8.7 (unused)—give to someone else 57.7 (unused)—keep at home until expired	[78]
	Saudi Arabia	All country	2018–2019	Pharmacy undergraduate or postgraduate students	An online website survey	464	15.0	89.0	-	22.0—give to someone else	[37]
	Saudi Arabia	Riyadh City	2019	Community pharmacists	The survey was distributed in person.	360	73.3 (liquid) 75.3 (solid) 74.2 (ointments and creams)—return to pharmaceutical distributor 15.5 (liquid) 16.1 (solid) 16.7 (ointments and creams) -putting in the medicines’ bin	1.7 (liquid) 3.6 (solid) 3.3 (ointments and creams)	4.4 (liquid) 1.9 (solid) 2.8 (ointments and creams)—toilet 3.6 (liquid) 1.1 (solid) 1.4 (ointments and creams)—sink	1.1 (liquid) 1.4 (solid) 1.1 (ointments and creams)—other	[38]
	Saudi Arabia	All country	2020	Participants from all areas of Saudi Arabia	An online website survey	924	6.0 (OTC) 10.0 (POM)	45.0 (OTC) 42.0 (POM)	6.0 (OTC) 7.0 (POM)	7.0 (OTC) 6.0 (POM)—donate to charity 18 (OTC) 14 (POM)—give to someone else	[79]
	Turkey	All country	2016	TGB company employees representing almost each geographic region of Turkey	An online survey	1121	34.0	33.9		32.9—bring to company’s drug-box	[80]
Europe	Ireland	Galway, Cork	2010–2011	Residents of Galway and Cork over the age of 18	Interview with people in the streets	398	28.0	51.0	14.0—toilet 29.0—sink	6.0—other	[81]
	Poland	All country	2015	Respondents from all country	An online survey	450	30.0	68.0		-	[8]
		Pomeranian district		Clients of the pharmacies over the age of 18	The questionnaires were distributed to clients by pharmacists.	635	35.7	35.0		-	
	Portugal	All country	2014	Household members over the age of 18	Face-to-face interviews	244	69.0	25.0	2.0	1.0—recycling depot 3.0—other	[48]
	Romania	Bihor Country	2014–2015	Clients of the five pharmacies (three urban and two rural pharmacies) over the age of 18	The questionnaires were distributed to clients by pharmacists.	771	0.7	95.3	-	4.0—throw away to other places	[47]
	Serbia	The South Backa District	2010	Household members, over the age of 18	Face-to-face interviews	230	4.5	80.3	7.6	6.6—burn 1.0—give to someone else	[41]
	Serbia	Novi Sad city	2011–2012	Residents of Novi Sad over the age of 18	Face-to-face interviews	383	4.4	82.8	2.9	1.8—burn 0.8—store 7.3—keep at home until expired	[82]
Australia	Australia	All country	2016	Residents over the age of 18	An online survey	4302	22.5	64.9	23.2	3.0—burn 6.0—bury 1.0—other	[83]

* depending on the dosage forms and class of the pharmaceuticals. OTC—Over-the-Counter. POM—Prescription Only Medicines.

## Data Availability

Not applicable.
